# Supplementary material for: Increased pupal temperature has reversible effects on thermal performance and irreversible effects on immune system and fecundity in adult ladybirds
Source: Commun Biol. 2023 Aug 12;6:838. doi: 10.1038/s42003-023-05196-0 (PMC10423239; doi:10.1038/s42003-023-05196-0)
Supplement: Supplementary file 2 — Supplementary Information [file 42003_2023_5196_MOESM2_ESM.pdf]

Increased pupal temperature has reversible effects on thermal performance and irreversible effects on immune system and fecundity in adult ladybirds

David N. Awde, Michal Řeřicha, Michal Knapp

Supplementary Materials

Table S1. Main and interaction effects of experiment variables on the chill comma recovery time (CCRt), heat knockdown time (HKDt), haemocyte concentration (haemocyte/µL), starvation resistance (longevity without food), time until the first clutch (days), and cumulative egg production of adult *Harmonia axyridis.*

Model number	Response variable	Experiment variables	df	χ2	p
1	Chill coma recover time	Live Mass	1	0.02	0.83
		Sex	1	0.73	0.39
		Temperature	2	55.13	**<0.001**
		Day	1	0.11	0.74
		Temperature * Day	2	16.11	**<0.001**
2	Heat knockdown time	Dry Mass	1	0.07	0.80
		Sex	1	1.87	0.17
		Temperature	2	13.76	**0.001**
		Day	1	1.08	0.30
		Temperature * Day	2	1.57	0.46
3	Haemocyte concentration	Sex	1	12.00	**<0.001**
		Temperature	2	72.19	**<0.001**
		Day	1	145.23	**<0.001**
		Temperature * Day	2	12.96	**0.002**
4	Starvation resistance	Live mass	1	62.94	**<0.001**
		Sex	1	16.30	**<0.001**
		Temperature	2	28.23	**<0.001**
5	Preovipositioning period	Live mass	1	1.57	0.21
		Temperature	2	2.95	0.23
6	Cumulative egg count	Live mass	1	0.90	0.34
		Temperature	2	1.20	0.55
		Day	1	1146.78	**<0.001**
		Temperature * Day	2	8.07	**0.02**

Table S2. Generalized linear mixed models used to assess the effects of developmental temperature and time since exposure on select traits of adult *Harmonia axyridis*. All models were run using the *glmmTMB* function from the *glmmTMB* package in RStudio (details in text).

Model number	Response variable	Model	Distribution
1	Chill coma recover time	~ Live mass + Sex + Temperature * Day + (1|Family)	Negative binomial
2	Heat knock-down time	~ Dry mass + Sex + Temperature * Day + (1|Family)	Negative binomial
3	Haemocyte concentration	~ Sex + Temperature * Day + (1|Family)	Negative binomial
4	Starvation resistance	~ Live mass + Sex + Temperature + (1|Family)	Gaussian
5	Preovipositioning period	~ Live mass + Temperature + (1|Family)	Gaussian
6	Cumulative egg count	~ Live mass + Temperature * Day + (Day|ID)	Negative binomial

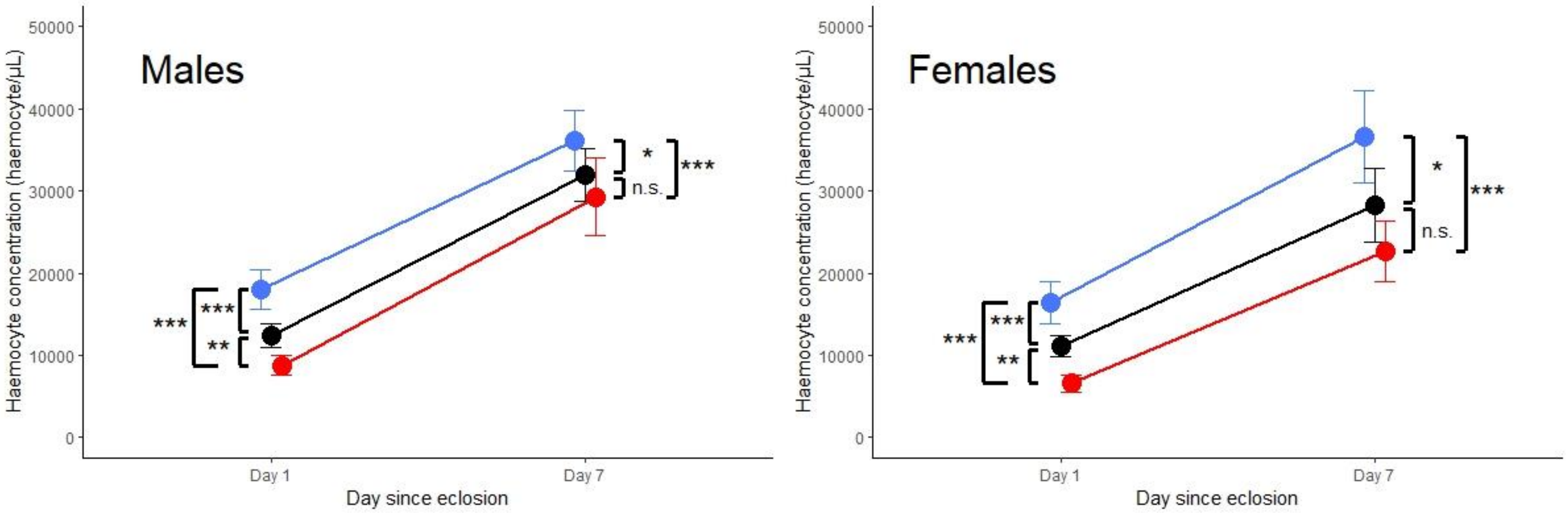

Figure S1. Effects of developmental temperature on haemocyte concentrations of males (left) and females (right) separately. Means ± 95% confidence intervals are plotted (* denote pairwise comparisons in which p <0.05, ** in cases that p <0.01, and *** in cases that p<0.001).

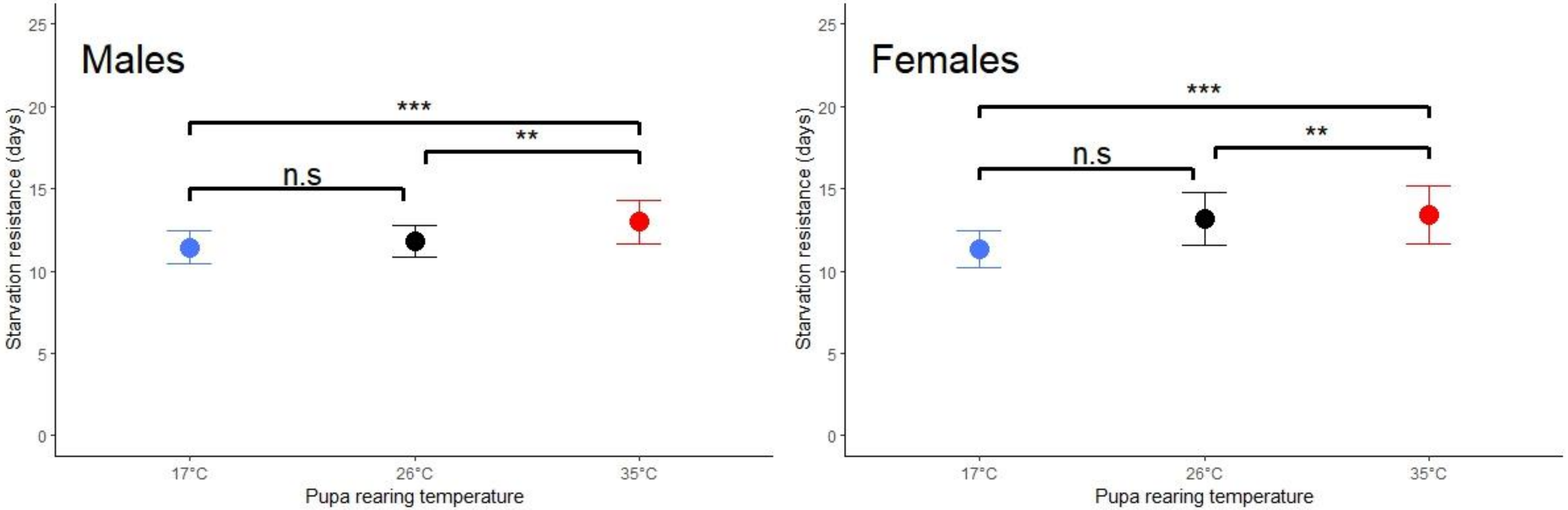

Figure S2. Effects of developmental temperature on starvation resistance of males (left) and females (right) separately. Means ± 95% confidence intervals are plotted (* denote pairwise comparisons in which p <0.05, ** in cases that p <0.01, and *** in cases that p<0.001).

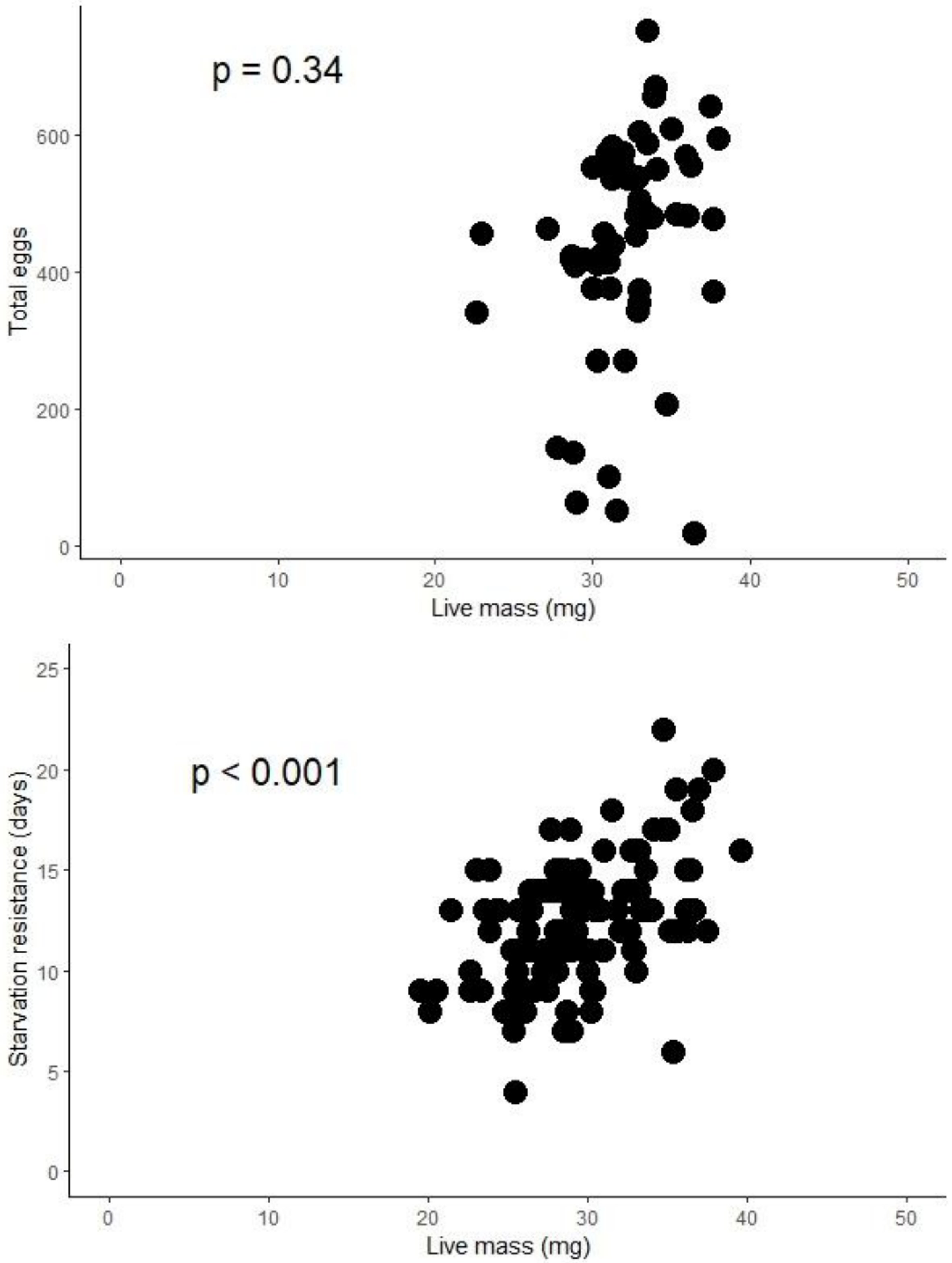

Figure S3. Size effects on cumulative egg production (top) and starvation resistance (bottom).
