# Supplementary material for: Increased pupal temperature has reversible effects on thermal performance and irreversible effects on immune system and fecundity in adult ladybirds
Source: Commun Biol. 2023 Aug 12;6:838. doi: 10.1038/s42003-023-05196-0 (PMC10423239; doi:10.1038/s42003-023-05196-0)
Supplement: Supplementary file 3 — Reporting Summary [file 42003_2023_5196_MOESM3_ESM.pdf]

nature portfolio

Corresponding author(s): Michal Knapp

Last updated by author(s): Jul 25, 2023

Reporting Summary

Nature Portfolio wishes to improve the reproducibility of the work that we publish. This form provides structure for consistency and transparency in reporting. For further information on Nature Portfolio policies, see our Editorial Policies and the Editorial Policy Checklist.

Statistics

For all statistical analyses, confirm that the following items are present in the figure legend, table legend, main text, or Methods section.

n/a	Confirmed	
☐	☒	The exact sample size (*n*) for each experimental group/condition, given as a discrete number and unit of measurement
☐	☒	A statement on whether measurements were taken from distinct samples or whether the same sample was measured repeatedly
☐	☒	The statistical test(s) used AND whether they are one- or two-sided<br>*Only common tests should be described solely by name; describe more complex techniques in the Methods section.*
☐	☒	A description of all covariates tested
☐	☒	A description of any assumptions or corrections, such as tests of normality and adjustment for multiple comparisons
☐	☒	A full description of the statistical parameters including central tendency (e.g. means) or other basic estimates (e.g. regression coefficient) AND variation (e.g. standard deviation) or associated estimates of uncertainty (e.g. confidence intervals)
☐	☒	For null hypothesis testing, the test statistic (e.g. *F*, *t*, *r*) with confidence intervals, effect sizes, degrees of freedom and *P* value noted<br>*Give P values as exact values whenever suitable.*
☒	☐	For Bayesian analysis, information on the choice of priors and Markov chain Monte Carlo settings
☒	☐	For hierarchical and complex designs, identification of the appropriate level for tests and full reporting of outcomes
☒	☐	Estimates of effect sizes (e.g. Cohen's *d*, Pearson's *r*), indicating how they were calculated

Our web collection on statistics for biologists contains articles on many of the points above.

Software and code

Policy information about availability of computer code

Data collection	No software was used to collect the data
Data analysis	All statistics and figures were performed and created in R and RStudio, version 4.2.1 and 2022.02.3, respectively. We used the function check_distribution from the package performance to assess the probability that a given variable belonged to a specific distribution. We used the glmmTMB function from the glmmTMB package to analyse CCRt, HKDt, haemocyte concentration, starvation resistance, cumulative egg production, and preoviposition period. The performance of each model was visually inspected using the check_model function from the performance package. We generated summary statistics of each model using the Anova (type "III") function from the Car package. Pairwise comparisons were performed with Tukey's tests using the lsmeans function from the lsmeans package. Figures were created using ggplot2.

For manuscripts utilizing custom algorithms or software that are central to the research but not yet described in published literature, software must be made available to editors and reviewers. We strongly encourage code deposition in a community repository (e.g. GitHub). See the Nature Portfolio guidelines for submitting code & software for further information.

Data

Policy information about availability of data

All manuscripts must include a data availability statement. This statement should provide the following information, where applicable:

- Accession codes, unique identifiers, or web links for publicly available datasets
- A description of any restrictions on data availability
- For clinical datasets or third party data, please ensure that the statement adheres to our policy

Raw data used to produce the figures and support the findings of this study are available in the Dryad Digital Repository DOI: 10.5061/dryad.1c59zw413

Research involving human participants, their data, or biological material

Policy information about studies with human participants or human data. See also policy information about sex, gender (identity/presentation), and sexual orientation and race, ethnicity and racism.

Reporting on sex and gender	This study did not involve human participants
Reporting on race, ethnicity, or other socially relevant groupings	This study did not involve human participants
Population characteristics	This study did not involve human participants
Recruitment	This study did not involve human participants
Ethics oversight	This study did not involve human participants

Note that full information on the approval of the study protocol must also be provided in the manuscript.

Field-specific reporting

Please select the one below that is the best fit for your research. If you are not sure, read the appropriate sections before making your selection.

☐ Life sciences ☐ Behavioural & social sciences ☒ Ecological, evolutionary & environmental sciences

For a reference copy of the document with all sections, see nature.com/documents/nr-reporting-summary-flat.pdf

Ecological, evolutionary & environmental sciences study design

All studies must disclose on these points even when the disclosure is negative.

Study description	In this study we assessed the effects of pupal temperature (17°C – normal temperature, 26°C – increased temperature, or 35°C – heat wave) and adult age after exposure effected adult Harmonia axyridis thermal stress tolerance, immune function, starvation resistance, and fecundity. Test subjects were individually sampled for only one response variable. For thermal tolerance, we aimed to analyse up to six individuals for each combination of variables (sex, parental pair, pupal temperature, and sampling time; n = 336. However, some parental pairs produced an insufficient number of offspring, resulting in 289 beetles measured for CCRT and 270 for HKDT. For immune function, We aimed to analyze four individuals for each combination of sex, parental pair, pupal temperature, and sampling time (1 day or 7 days; n =336 ladybirds). However, problems sampling several individuals (e.g., haemolymph coagulation) resulted in a smaller dataset of 308 beetles. For starvation resistance, we sampled 126 individuals, i.e., three individuals per combination of sex (2), parental pair (7), and pupal temperature (3). For fecundity, we sampled 63 individuals.
Research sample	The research samples in this study were lab-reared ladybirds, Harmonia axyridis. This species is a widespread invasive insect around the globe. The parental generation for this population was sourced from České Budějovice, Czech Republic (48.9794344 N, 14.4451989 E), and is viewed as a reprentative population for most of central Europe.
Sampling strategy	Sample sizes were chosen to maximize our ability to make comparisons across variables (parental generation, pupal temperature, day since exposure, and sex), while also maintaining a manageable source population given laboratory resources.
Data collection	Raw data was collected by Michal Knapp and Michal Řeřicha using direct laboratory measurements of starvation resistance, fecundity (number of eggs produced), chill coma recovery time, heat knock down time, haemocyte concentration (using optical microscope equipped with Burker chamber).
Timing and spatial scale	5/2014-8-2014
Data exclusions	No data was excluded from the analyses
Reproducibility	All details are described in the manuscript.

Reproducibility

Ladybird rearing and experimental setup

Individuals assayed in our experiment were the offspring of field-collected adults from České Budějovice, Czech Republic (48.9794344 N, 14.4451989 E). Seven parental pairs were placed into separate Petri dishes (9 cm in diameter) containing folded paper strips as substrate for egg laying (Figure 4). Parental pairs were kept in a climatic chamber (Sanyo® MIR-155) at 26 °C, relative humidity of 70%, and long day photoperiod (16L:8D). Ladybirds were provided with fresh pea aphids and water ad libitum daily. Eggs from each parental pair were collected, each clutch placed into separate Petri dishes, and kept under the same conditions as parental pairs. After hatching, larvae were separated into smaller groups of 4-5 individuals, placed into new Petri dishes, and provided with fresh pea aphids and water ad libitum. The last instar larvae were placed individually into new Petri dishes, fed until the prepupal stage, and then checked twice a day to collect fresh pupae as soon as possible. Petri dishes with fresh pupae were immediately distributed among three climatic chambers set to one of three thermal regimes: normal temperature (17°C), increased temperature (26°C), or heat wave (35°C; Figure 4). Pupae were checked twice a day (morning and evening) to collect freshly eclosed adults. The pupal stage was selected because of its lack of mobility, and therefore its inability to escape such high stressful temperatures under natural conditions.

New adults were moved to the 26°C climatic chamber and provided with pea aphids and water ad libitum (Figure 4). After 24 hours, the first subset of adults was used to measure haemocyte concentration and thermal performance. Seven days after eclosion, the same variables were measured for the second subset of adults. The third subset of ladybirds was used to assay ladybird longevity without food. Finally, the fourth subset was used to assay fecundity (e.g., preoviposition period, number of clutches, etc.).

Thermal performance

To investigate high- and low-temperature performance of ladybirds subjected to various pupal temperatures, we measured chill coma recovery time (CCRt;53,54) and heat knockdown time (HKDt;55). To measure CCRt, sets of six ladybirds in separate Petri dishes were exposed to -4°C in a climatic chamber for 120 minutes and then transferred to a climatic chamber set to 26°C for recovering. Beetles were turned onto their dorsum and time until the first coordinated movement was measured (e.g., flipping to their ventral side). Experimental ladybirds were stimulated to move by gentle teasing with an entomological pin every fifteen seconds to identify beetles that remained motionless despite being already able to move. To measure HKDt, sets of 12 individuals (in few cases less) were placed in clean Petri dishes (without food and water) in a climatic chamber set to 42°C. Ladybirds were checked every 15 minutes using a webcam placed inside the chamber. Individuals that had become immobile or dead (reached their HKDt) were easily identified as motionless, typically with spread wings. We aimed to analyse up to six individuals for each combination of variables (sex, parental pair, pupal temperature, and sampling time; n = 336 ladybirds). However, some parental pairs produced an insufficient number of offspring, resulting

in 289 beetles measured for CCRT and 270 for HKDT (raw data available in the Dryad digital repository[56]). As a measure of body size, we recorded the live body mass after eclosion of beetles assayed for CCRt using a Sartorius balance with a precision of 10−4g. All beetles measured for HKDt were dried for 48 hours at 42°C after the HKDt assay and their dry mass recorded.

Haemocyte concentration

Haemolymph was collected via puncture sampling[57]. Haemolymph was collected using a glass microcapillary (Hirschmann, Germany) and measured using a digital calliper with a precision of 0.01 mm. Collected haemolymph (~1µl) was immediately diluted (100× dilution) in anticoagulant buffer (Phosphate-buffered saline: 137 mM NaCl, 2.7 mM KCl, 10 mM Na_2HPO_4, and 1.8 mM KH_2PO_4), and the total haemocyte concentration was recorded immediately using a Bürker chamber under a Carl Zeiss Primo Star microscope (set to 100× magnification). We aimed to analyze four individuals for each combination of sex, parental pair, pupal temperature, and sampling time (1 day or 7 days; n =336 ladybirds). However, problems sampling several individuals (e.g., haemolymph coagulation) resulted in a smaller dataset of 308 beetles[56].

Starvation resistance

We indirectly tracked the whole-body effects of developmental thermal acclimation on ladybird physiology by assessing longevity under a physiologically stressful condition, food deprivation. Ladybirds used to investigate the effects of pupal temperature on starvation resistance (longevity without food) were placed in Petri dishes with watered pieces of cotton wool and moved to a climatic chamber set to 26°C. Survival of these ladybirds was checked daily, and new water was added ad libitum. In total, starvation resistance was recorded for 126 individuals, i.e., three individuals per combination of sex (2), parental pair (7), and pupal temperature (3). Their live body mass after eclosion was measured using a Sartorius balance with a precision of 10−4 g since body mass is known to influence starvation resistance in ladybirds[58].

Fecundity

Finally, we evaluated the fitness consequences of thermal acclimation during pupation by tracking the cumulative egg production and days until the first clutch of females after eclosion. We recorded egg production each of 28 days after eclosion. Experimental pairs (n = 63 males and females) were established from a combination of individuals that eclosed on the same day and originated from different parental pairs. Each experimental pair was placed in one Petri dish for mating, placed in a climatic chamber set to 26°C, relative humidity of 70%, and long day photoperiod (16L:8D; same as the parental generation), and provided with pea aphids and water ad libitum. Every day folded paper strips in Petri dishes were changed and the total number of eggs was counted. Note that ladybirds typically lay eggs on the underside of leaves, which is mimicked by folded paper

298 strip. Live body mass was measured for each experimental female after eclosion.

Randomization	Offspring from one parental pair were evenly allocated to temperature treatments (17, 26 or 35 °C) - individuals were drawn completely at random.
Blinding	Blinding was applied in form of ID codes that didn't included direct treatment information.

Did the study involve field work? ☐ Yes ☒ No

Reporting for specific materials, systems and methods

We require information from authors about some types of materials, experimental systems and methods used in many studies. Here, indicate whether each material, system or method listed is relevant to your study. If you are not sure if a list item applies to your research, read the appropriate section before selecting a response.

Materials & experimental systems

n/a	Involved in the study	
☒	☐	Antibodies
☒	☐	Eukaryotic cell lines
☒	☐	Palaeontology and archaeology
☐	☒	Animals and other organisms
☒	☐	Clinical data
☒	☐	Dual use research of concern
☒	☐	Plants

Methods

n/a	Involved in the study	
☒	☐	ChIP-seq
☒	☐	Flow cytometry
☒	☐	MRI-based neuroimaging

Animals and other research organisms

Policy information about studies involving animals; ARRIVE guidelines recommended for reporting animal research, and Sex and Gender in Research

Laboratory animals	The study used the insect (ladybird) Harmonia axyridis
Wild animals	The study did not involve wild animals.
Reporting on sex	Sex-based analyses were performed.
Field-collected samples	Ladybirds were kept in Petri dishes (9 cm in diameter) containing folded paper strips. Petri dishes kept in a climatic chamber (Sanyo® MIR-155) at 26 °C or their assigned treatment condition, relative humidity of 70%, and long-day photoperiod (16L:8D). Ladybirds were provided with fresh pea aphids and water ad libitum daily
Ethics oversight	No ethical approval or guidance was required to work on these insects, ladybirds.

Note that full information on the approval of the study protocol must also be provided in the manuscript.
